# Integrating community voices in the research continuum: Perspectives on a consultation service

**DOI:** 10.1017/cts.2023.600

**Published:** 2023-07-28

**Authors:** Crystal D. Evans, Joy P. Nanda, Pamela Ouyang, Lee Bone, Samuel Byiringiro, Cyd Lacanienta, Roger Clark, Christine Weston, Hae-Ra Han, Mia Terkowitz, Barbara Bates-Hopkins, Panagis Galiatsatos, Ashley Jingzhi Xu, Sarah Stevens, Cheryl R. Himmelfarb

**Affiliations:** 1 Institute for Clinical and Translational Research (ICTR), Johns Hopkins University, Baltimore, MD, USA; 2 Community Research Advisory Council, The Johns Hopkins ICTR, Baltimore, MD, USA; 3 The Johns Hopkins University School of Medicine, Baltimore, MD, USA; 4 The Johns Hopkins University Bloomberg School of Public Health, Baltimore, MD, USA; 5 The Johns Hopkins University School of Nursing, Baltimore, MD, USA

**Keywords:** Translational research, community engagement, community advisory board, consultation service, research continuum

## Abstract

The Community Research Advisory Council (C-RAC) of the Johns Hopkins Institute for Clinical and Translational Research was established in 2009 to provide community-engaged research consultation services. In 2016–2017, C-RAC members and researchers were surveyed on their consultation experiences. Survey results and a 2019 stakeholder meeting proceeding helped redesign the consultation services. Transitioning to virtual consultations during COVID-19, the redesigning involved increasing visibility, providing consultation materials in advance, expanding member training, and effective communications. An increase in consultations from 28 (2009–2017) to 114 (2020–2022) was observed. Implementing stakeholder-researcher inputs is critical to holistic and sustained community-engaged research.

## Introduction

Integrating community voices is integral to successful community-engaged research (CEnR) [1–7]. By developing strategies from community voices, researchers can orient their research topics and design studies that are most beneficial to patients and communities [8–11]. The Clinical Translational Science Award (CTSA) program mandates strong community-academic partnerships to build researchers' capacities to advance CEnR [10–15].

The Johns Hopkins the Institute for Clinical and Translational Research (ICTR) was established in 2007 to address health equity issues among Maryland residents. Through its Community and Collaboration Core, the ICTR engages community partners, stakeholders, and researchers to codesign, implement, evaluate, and disseminate clinical and translational research. Within the Community and Collaboration Core, the Community Research Advisory Council (C-RAC) provides consultation services for researchers who request this service. From 2009–2016, the C-RAC consultation service provided researchers with a vehicle to strengthen community-academic partnerships while enhancing funding prospects. The service program also provided C-RAC members opportunities to become advisory board members for some of the studies consulted. Using post-consultation surveys in 2016 and 2017, the C-RAC analyzed data from both researchers and C-RAC members to learn about their experience with the consultation process. Results from these surveys informed an ICTR-wide stakeholder retreat meeting in 2019, which informed goals and objectives to redesign and build a framework to sustain the CEnR consultation process [9].

In particular, transitioning to virtual consultation engagement due to the COVID-19 pandemic, the redesigned consultation framework has been implemented since 2020. This report reflects refinements that were made by C-RAC using the 2016–2017 post-consultation survey results and directives from the 2019 retreat [9].

## Initial Consultation and Post-Consultation Steps

### C-RAC Composition

The C-RAC is composed of 22 members representing patients/research participants, community-based organizations, neighborhood associations, health systems, and historically black colleges and universities. Membership included twelve Blacks/African American, six White, two Asian, and two Hispanic individuals. Fifteen were female, six were male, and one was nonbinary; with mean age of 55 years. Sixteen members have served 5–7 years. Ten members were Johns Hopkins affiliates.

### Researcher Composition

Researchers requesting consultation services represented disciplines including cardiology, pulmonary and critical care, human genomics, cancer, biomedical informatics, emergency medicine, infectious disease, and obstetrics-gynecology. The researchers included tenured and nontenured faculty from Johns Hopkins University.

### The Original C-RAC Consultation Steps

**Figure 1.**
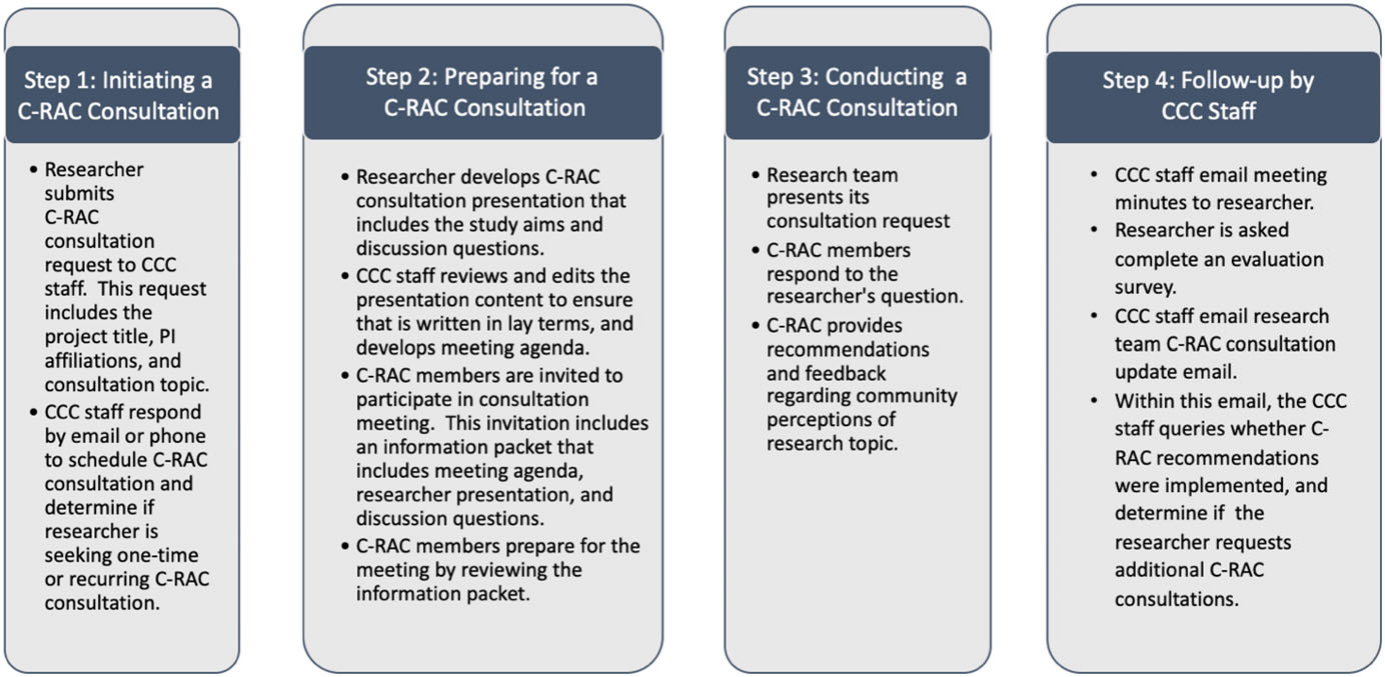
The original four steps of the Community Research Advisory Council (C-RAC) consultation process.

Figure 1 displays the four original consultation steps established in 2009: (1) Researcher-initiated request, (2) Provision of prepared consultation materials to C-RAC members, (3) Consultation meeting and feedback, and (4) Technical support team’s post-consultation meetings with researchers to evaluate implementation of C-RAC recommendations. The consultation service was originally marketed through word-of-mouth, referrals, and Johns Hopkins IRB.

From 2009 to 2016, C-RAC provided 28 consultations to 25 researchers: Requests were made for 13 pre-grant submissions and 15 post-award projects. The IRB required community input from four projects.

Overall, 13 researchers requested C-RAC input on study design, six on recruitment and retention, two on data collection strategies, 8 on community advisory board formation, seven on forming partners, and five on disseminating information. The above requests overlapped among researchers.

### Post-Consultation Surveys

Post-consultation surveys were administered between 2016 and 2017 to inform the consultation redesign. Qualtrics-based surveys were emailed to the 25 researchers and 22-C-RAC member participants from the 2009–2016 consultations. Fourteen of 25 researchers and 19 of 22-C-RAC members responded. Responses were exported to databases for analysis of qualitative and quantitative data separately. Frequency distribution was generated for quantitative data. The qualitative survey responses were reviewed and categorized by theme by the authors of this manuscript and other participants at the ICTR stakeholder retreat. Consensus-based themes that were generated are described in the implementation section of this report [9].

Table 1 displays survey structured questions and results. The researcher survey consisted of 10 questions: three structured (nominal, discrete, ordinal) and seven open-ended, including reasons for consultation requests and satisfaction with the services. The C-RAC member survey included 16 questions: thirteen structured and three open-ended queries. The structured questions included demographics and the consultation experience of the C-RAC member. The open-ended queries generated improvement strategies for consultation services.

**Table 1.** Survey responses to structured questions

Researcher Experience with Consultation Services					
	**Researcher Experience Questions**	**Response Frequencies**			
**1**	How did you find out about the Community Engagement Consultation Service? (Please be specific)	Open-ended			
**2**	Who provided the consultation? (Provide Name)	Open-ended			
**3**	What was the purpose of your consultation? (Check ALL that apply) **(*n* = 14)** **Top 3**	Feedback before grant submission = 8	Need for patient advisory board = 6		Identifying community partners = 6
**4**	How useful was the consultation in meeting your needs? **(*n* = 14)**	Somewhat useful = 2	Very useful = 5		Extremely useful = 7
**5**	Please elaborate on your answer above. What made the consultation especially helpful, OR, what could have made the consultation more helpful?	Open-ended			
**6**	What were some of the most valuable recommendations or suggestions that you took away from the consultation?	Open-ended			
**7**	Were you able to incorporate any of these recommendations into your research? Why or why not? Please explain **(n = 14)**	Yes = 13			
**8**	How likely would you be to recommend this consultation service to others? **(*n* = 14)**	Likely = 4		Very Likely = 10	
**9**	What can we do to improve the consultation service?	Open-ended			
**10**	Any Additional comments/suggestions	Open-ended			
**C-RAC Members Experience with Consultation Services**					
**1**	Have you ever participated in a C-RAC Research Review? **(*n* = 19)**	Yes = 14	No; but would like to = 4		No; do not want to = 1
**2**	About how many C-RAC research reviews have you participated in? **(*n* = 14)**	1–3 = 6	4–6 = 3		7 or more = 4
**3**	How do you feel about the number of research reviews that you are asked to contribute to? **(*n* = 13)**	Can do more than current = 9 Just the right amount = 4			
**4**	How prepared do you feel to conduct the research reviews? **(*n* = 13)**	Somewhat prepared = 10		Very prepared = 3	
**5**	How confident do you feel in your ability to contribute to the research reviews? (***n* = 13)**	Somewhat confident = 6		Very confident = 8	
**6**	How confident are you that the researcher will use the advice that C-RAC members provide to him/her? (***n* = 14)**	Somewhat confident = 9		Very confident = 4	
**7**	Have you participated in research reviews about the following topics? (Check all that apply) **Top four topics** (*n* = 14)	Diabetes = 8	Heart Disease = 6	Asthma = 6	Alzheimer’s = 6
**8**	What do you most like about the C-RAC Research Review?	Open-ended			
**9**	List two things we can do to improve the C-RAC process	Open-ended			
**10**	People participate in C-RAC Research Reviews for various reasons. Please check all of the reasons that you participated: (Check all that apply) **Top four reasons (*n* = 14)**	– To inform researchers about concerns of people living in my community = 12* – To learn about the research process = 11 – To share what I learn with my community = 10 – To hear about what research is done in my community = 9			
**11**	We would like to develop research trainings for the C-RAC and other community boards. What topics would you like to learn more about? (Check all that apply) **Top four reasons (*n* = 14)**	Research Methods (Different ways of doing research) = 13 Getting research results back to my community = 13 Developing research questions = 11 Preparing a grant proposal = 11			
**12**	How often should we offer these trainings? **(*n* = 18)**	1–2 times/year = 8		3–4 times/year = 10	
**13**	How do you think trainings should be offered? **(*n* = 19)**	Both in-person and online = 16		In-person only = 3	
**14**	Are you a member of the C-RAC? **(*n* = 18)**	East Baltimore Chapter = 7	Bayview Chapter = 4		Both Chapters = 7
**15**	How long have you been a member of the C-RAC? **(*n* = 18)**	<1 year = 4	1–3 years = 8		≥4 years = 6
**16**	Additional Comments	Open-ended			

### Researcher Satisfaction with Consultation Services

Twelve researchers found the consultation services very or extremely useful in meeting their research needs and 13 reported incorporating C-RAC recommendations into their research. All 14 researchers responded that they would likely or very likely recommend the consultation service to the research community.

Most researchers learned about the consultation service through word-of-mouth, other faculty and staff involved with ICTR, and external sources (e.g., PCORI). Most common reasons for seeking consultation were: (1) feedback on the project prior to grant submission; (2) assistance in developing a community advisory board; (3) identification of community partners. Researchers also noted the extreme helpfulness of technical staff.

Gaining access to diverse stakeholders with “lived and professional experience” helped inform study design, simplify survey instruments, and identify recruitment strategies.

Researchers incorporated C-RAC recommendations into their studies by modifying consent forms, increasing participant reimbursement rate, developing websites to disseminate study results, and recruiting and training study participants to serve as research ambassadors.

### C-RAC Member Experience with Consultation Services

Fourteen C-RAC members reported participating in at least one consultation. Most frequent consultation topics were diabetes (*n* = 8), heart diseases (*n* = 6), asthma (*n* = 6), and Alzheimer’s disease (*n* = 6). C-RAC consultations provided a forum for members to inform researchers about the concerns of people living in their communities (*n* = 12), learn about the research process (*n* = 11), share research information with community members (*n* = 10), and hear about research relevant to their communities (*n* = 9). Thirteen C-RAC members were interested in increasing the number of consultations.

Ten C-RAC members were somewhat prepared for the consultations whereas six were very confident in their ability to contribute to the consultation process. Nine were also very confident of researchers using C-RAC feedback to improve their research.

To improve the consultation process, thirteen C-RAC members wanted a better understanding of research methods and getting research results back to the community. Developing research questions (*n* = 11) and preparing grant proposals (*n* = 13) were other unmet training needs for C-RAC members. The frequency of these pieces of training could be 3–4 times (*n* = 10) or 1–2 times a year (*n* = 18). via both virtual and in-person venues.

**Figure 2.**
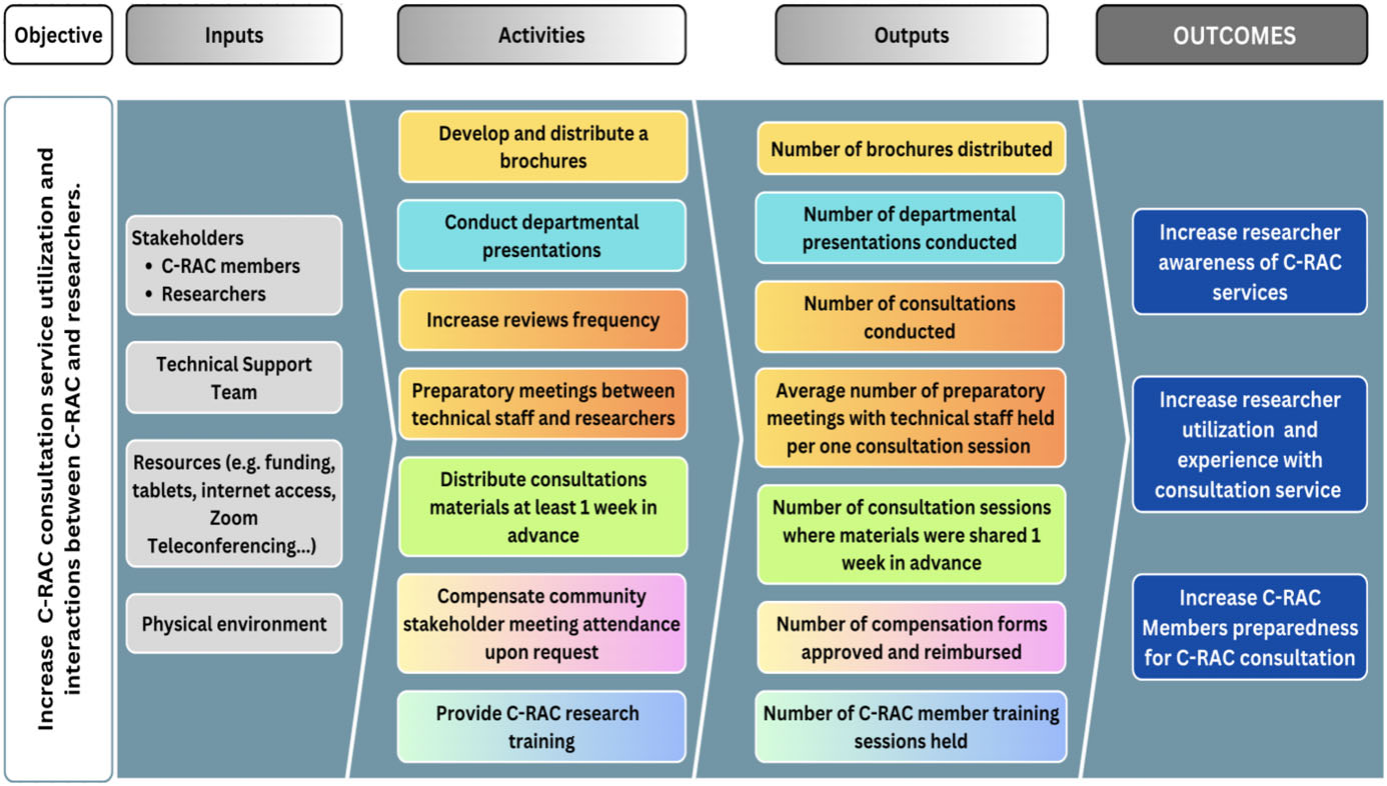
The logic model as a framework for refined Community Research Advisory Council (C-RAC) consultations.

The above results informed the 2019 strategic planning meeting comprising C-RAC members, community organizations, researchers, and the ICTR. Objectives for redesigning consultation services were formulated at this retreat. The Logic Model in Fig. 2 provided the framework to achieve those objectives.

## Implementation of Recommendations

### Adapting to the COVID-19 Pandemic

In 2020, the COVID-19 restrictions and increased consultation requests necessitated transitioning from in-person to virtual consultation meetings from monthly to weekly, respectively. Additionally, enhanced technical support provided tablets, internet access, and virtual meeting platforms to enable community members with limited access.

### Implementing Feedback on Improving the Consultation Service

The qualitative survey responses informed ways to improve the service after being reviewed and categorized by the authors and the C-RAC. Three themes emerged as follows:

1. **Increased visibility and capacity to promote CEnR practice:** There was consensus to increase the number of consultations through increased visibility and marketing. Accordingly, the service was marketed through presentations and Community and Collaboration Core website placement. Thirteen presentations at six scientific conferences and distribution of over 500 brochures at stakeholder events were useful marketing strategies. Additionally, C-RAC partnered with NIH-funded trainee (TL-1) program to mentor the next generation of researchers in CEnR practice [19]. Since 2020, three cohorts of 36 trainees have participated in C-RAC consultations [19]. The C-RAC also served as the advisory council for a Cardiovascular Study, COVID-19 Health Literacy research, and a COVID-19 testing study.
2. **Training:** Both researchers and C-RAC members recommended that members receive training to strengthen collaboration with researchers, including understanding of research methods and human subject protection. Between 2020 and 2022, C-RAC organized twelve training sessions on CEnR Health equity, Bioethics, Dissemination of Findings, Diversity and Inclusion, and Manuscript Development. In 2021, fourteen C-RAC members completed the Office of Human Research Protections online training certification.
3. **Increased communication:** Both groups indicated the need for instituting timely and effective communication (e.g., providing all consultation materials in advance) to improve preparedness before, and active participation during consultation sessions. C-RAC members also requested that researchers provide feedback as to whether their suggestions were implemented. In response, the number of consultations where materials were provided in advance of the meeting had more than doubled, and an increase from 3 to 45 researchers returning to C-RAC meetings to discuss implementation of recommendations was observed.

Overall, the recruitment of staff trained in CEnR practice facilitated the consultation process, and the C-RAC, by implementing a weekly schedule, conducted 114 consultations in 2020-2022.

### Redesigned Consultation Service

Two additional steps were incorporated into the original four-step process: (1) Formalizing iterative interactions between technical staff and researchers to clarify the needs of the consultation and (2) Post-consultation follow-up with researchers to determine the status of their research (e.g., achieving funding or recruitment goals) and the extent to which C-RAC recommendations were implemented. To achieve these steps, a dedicated staff member was assigned to coordinate the consultation service and to work closely with the researcher and C-RAC.

## Discussion and Conclusion

Our redesigned community-engaged consultation services have improved the efficiency of the research (e.g., timely reviews and dedicated support structure).

Reports on how feedback from community members and researchers was used to refine the consultation process are limited [16,17]. Enhancing technical support, increasing the visibility and consultation frequency, providing research training, and refining the consultation protocol have strengthened the consultation process holistically.

The C-RAC consultation process is in alignment with reports on overall satisfaction with community- consultations [7,10,14,16,17]. However, improvements made in response to feedback from researcher and community are insufficiently documented. Our efforts reinforce the significance of integrating community voices in improving the consultation process. These processes included: (1) advanced provision and review of consultation materials (2) bidirectional feedback on the implementation of C-RAC recommendations, (3) dedicated staff to help the researcher navigate the consultation process, and (4) training and capacity building for the C-RAC on research methods, ethics, human subject’s protection. The implementation of these changes during COVID-19 showed evidence of a noticeable increase in consultations that included COVID-19 (*n* = 19) and other research projects (*n* = 95). Increased marketing may have also sustained the C-RAC-researcher partnership.

### Limitations and Future Directions

We encountered setbacks while striving to optimize the consultation process. A methodical baseline and follow-up would have helped evaluate the implementation of recommendations. In actuality, the unanticipated increase in utilization of the C-RAC consultation services during COVID-19 limited our ability to formally evaluate the implementation in a timely manner. A comprehensive evaluation of the service following the changes described here is underway.

The low response rate to the researcher’s post-consultation survey may have biased our interpretation. However, the consensus among researchers at the strategic planning retreat was that the consultation process had accelerated a timely CEnR process, albeit a few step-wise improvements were needed. Implementation of the reengineered steps and preliminary assessments not only reflects an iterative bidirectional communication between C-RAC and researchers but also shows promise of increased participation in follow-up among these integral partners.
